# Form and Nature of Accidents Occasioned by the Eruption of Wisdom Teeth

**Published:** 1883-10

**Authors:** 

**Affiliations:** Paris.


ARTICLE IV.

### FORM AND NATURE OF ACCIDENTS OCCASIONED BY THE ERUPTION OF WISDOM TEETH.

BY DR. MAGITOT, PARIS.

Considered in a general manner, the accidents occasioned by the eruption of a wisdom tooth are very numerous.

A methodical division of the phenomena is not an easy thing, and very often an accident of a certain nature at the first appearance modifies itself to pass into and through other successive forms.

However, as a classification is necessary to the description, we have adopted the following:

1. Inflammatory accidents, subdivided into accidents of the mucous membrane, and accidents of the bony structures.

2. Nervous accidents, pain in the nerves, troubles in organs of some special sense, and reflex phenomena.

3. Organic accidents, which include the follicular cysts of the wisdom teeth, the odontomata, and new formations.

First, inflammatory accidents.

*Mucous accidents.*—The mucous accidents connected with the eruption of wisdom teeth are extremely frequent. They commence with a simple irritation of the gums and finish with an abscess, ulceration or gangrene. Sometimes, however, the local accident is isolated, sometimes there is a complication of disturbances of the neighboring parts more or less intense.

In all cases this mucous form of accidents is, of many, the most common, for in the statistics given by Dr David of seventy-five accidents due to this eruption, it represents a proportion of seventy observations, which gives in the sum of various accidents in the proportion of about 93 per cent.

In the most simple cases the mucous membrane of the region of the wisdom teeth is merely lifted, moderately swollen and tender. The accident, without passing unperceived, causes only a slight pain, and the tooth shortly overcoming this obstacle, appears above the gum in the midst of some shreds having about the aspect of proud flesh. The pressure of these shreds, which remain for some time on the chewing surface of the tooth, causes invariably the formation of certain pouches, which become filled with foreign matter and debris of food, and thus become true centres of the production of caries.

It is by this process that they cause so premature injuries to wisdom teeth to such a degree that some inattentive observers have asserted that wisdom teeth often erupt in a carious condition.

In a more pronounced stage of the inflammation of the gums, the portions of mucous membrane lying upon the tooth are the seat of a true abscess, at the centre of which the tooth itself is found, which remains thus imprisoned without communication with the exterior. It is to this form of accident that Chassaignac has given the name, *encystment* of the wisdom tooth, to distinguish it from the preceding form, which he calls encasing.

This distinction, which seems a little too fine, is in other respects wholly artificial; for the first form ordinarily blends with the other, when, after the spontaneous or intentional opening of the abscess, the enclosed tooth finds itself in communication with the outside.

The phlegmonous form consists of a true follicular abscess, and in this case the local accident most commonly extends itself to the neighboring parts. Sometimes it is an inflammation of the gums, which extends forward along the border of the gums and sometimes even to the median line. Sometimes it is an inflammation of a gland, whose particular character is that of extreme persistency as long as the cause remains unknown. Toirac mentions a very remarkable observation concerning it by Dr. Fiard. From the glands, the inflammatory process extends itself to the soft palate and to the pharynx, giving place to an equally obstinate inflammation.

Be it as it may, this form of accidents is altogether peculiar for the inferior and superior wisdom teeth.

For the inferior jaw, the simple eruption of a tooth, otherwise normal as to volume and direction, may be its cause, while in the upper jaw it produces only that condition when the wisdom tooth is directed abnormally either outside toward the cheek or backwards toward the anterior part of the palate. It is, moreover, generally to this form that the troubles confine themselves, as we have remarked. To the phlegmonous form which we were indicating, and now and then also to a simple inflammation of the gums, the ulcerous state often succeeds. One sees them on a level with the torn shreds of the mucous membrane, or, as a consequence of the opening of the follicular abscess, some irregular ulcerations with grayish bottom, covered with shreds of epithelium, which give the aspect described under the name of ulcero-croupous stomatitis.

The seat of these ulcerations is the region of the border of the gum around it, quite often it is the mucous membrane of the cheek ; more rarely the seat of the ulceration is on the tongue, when the wisdom tooth has a direction inwards. In every case the ulcerous variety is that which we have indicated elsewhere in speaking of the inflammation of the gums properly, as representing for a certain number of authors, and for ourselves, the true nature of ulcero-croupous stomatitis as occurring among the soldiers, and in general among young subjects at the age of the eruption of the wisdom teeth.

The mucous accidents of wisdom teeth comprise, then, as one sees, three varieties : the simple inflammation of the gums, the phlegmonous inflammation of the gums, and the ulcerous variety.

But this is not all. In these three varieties, more especially in the last two, there are produced ordinarily some complications in the neighboring parts. The most frequent is the inflammation of the sub-maxillary gland. It appears almost infalliably when the local inflammatory phenomena take a notable intensity of a certain duration. This adenitis, particularly persistent in the ulcero-croupous form which has been described as occurring among young soldiers as the consequence of the pressure of the collar, or other special cause, appears in our opinion as belonging to the series of accidents of the wisdom teeth. This grandular inflammation, considered as a complication of an accident of the mucous membrane, will have, nevertheless, for its exclusive seat, the sub-maxillary glands for the inferior jaw and the parotidal glands for the superior. The cervical glands themselves become swollen only when the morbid phenomena have invaded the bony tissues of the jaws. From grandular swelling to inflammation properly there is only step, and this new complication is very frequent. Here inflammation appears under the different forms which we have designated as simple œdema, circumscribed abscess, and diffused abscess, according to the extent of the original injury. We may say that in an accident of the mucous membrane the œdematious inflammation is the most common complications.

The phlegmonous form belongs to the cases of severe inflammation of the mucous membrane, or to those particular cases in which an inferior wisdom tooth finds itself enclosed in the soft parts of the cheek, where it determines ulcerations, indurations and fungous growths, in the midst of which the tooth may be found encased.—*Le Progress Dentaire*. L. B. BROOKS,—*New England Journal of Dentistry*.

